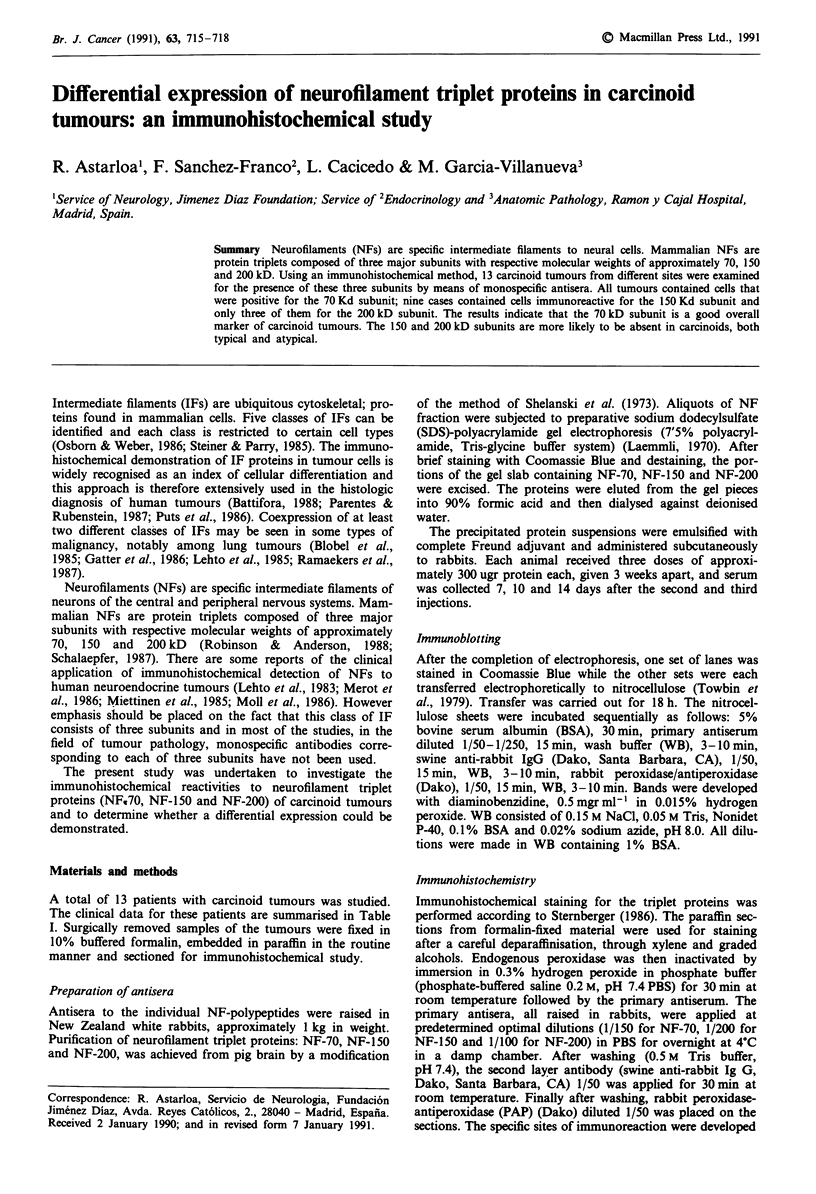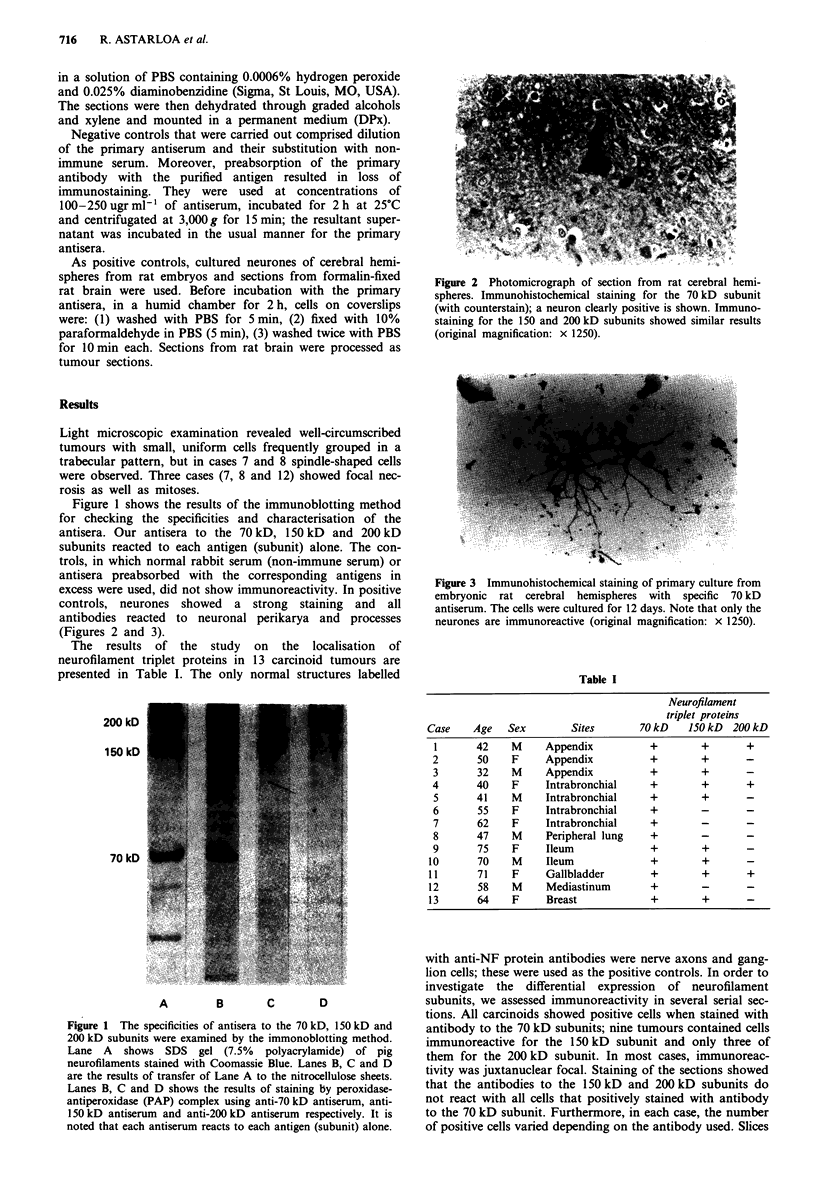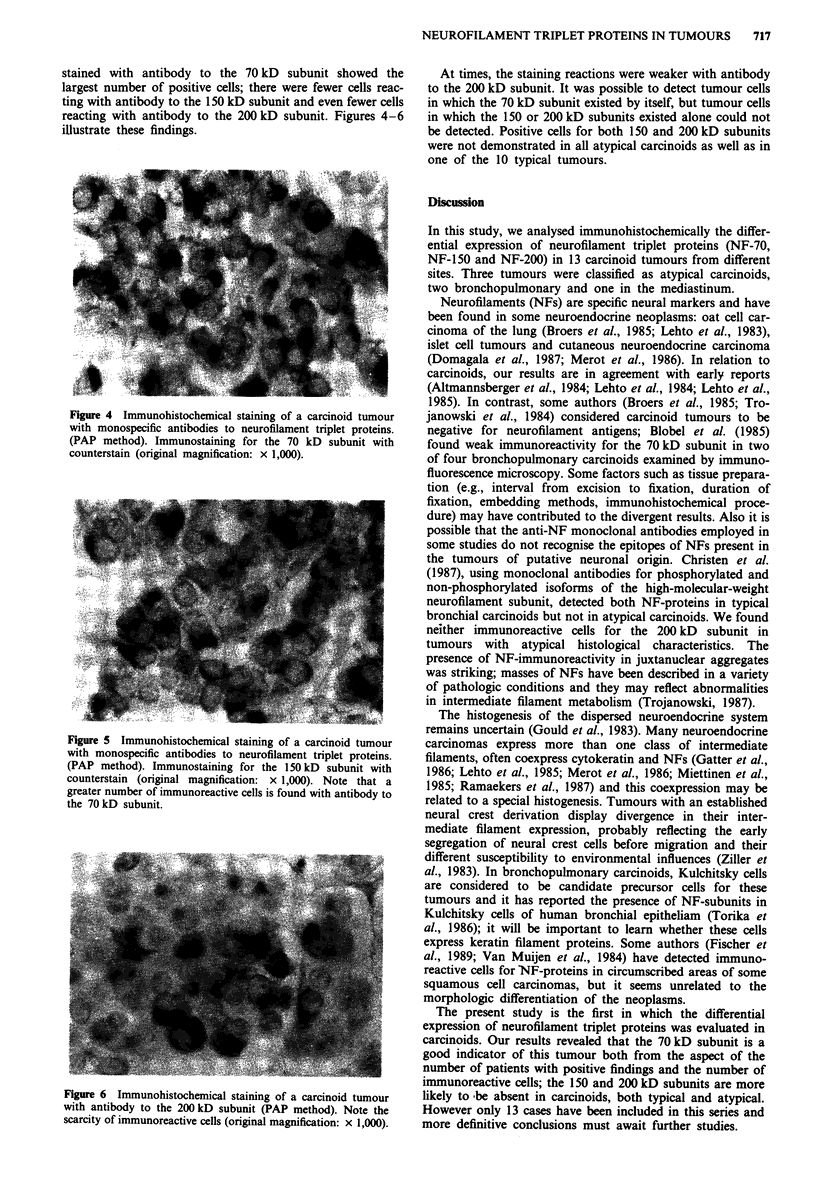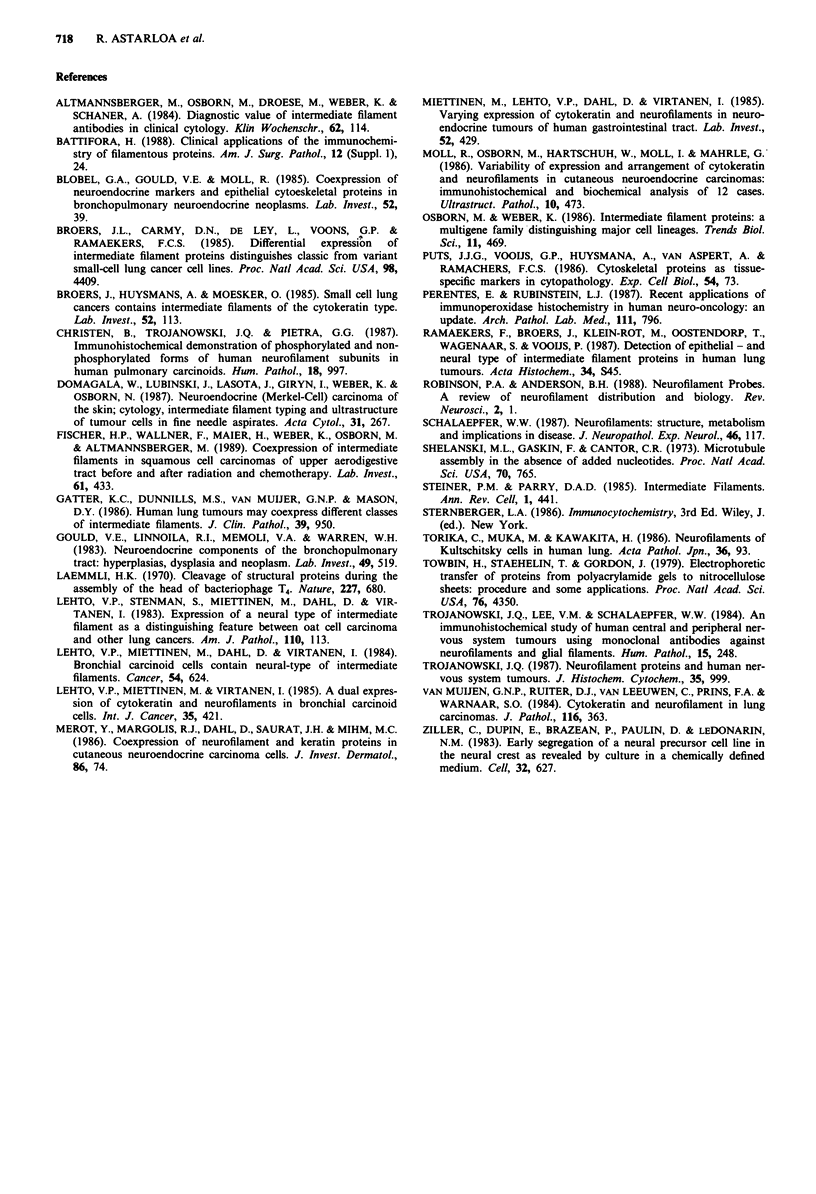# Differential expression of neurofilament triplet proteins in carcinoid tumours: an immunohistochemical study.

**DOI:** 10.1038/bjc.1991.161

**Published:** 1991-05

**Authors:** R. Astarloa, F. Sánchez-Franco, L. Cacicedo, M. García-Villanueva

**Affiliations:** Service of Neurology, Jiménez Díaz Foundation, Madrid, Spain.

## Abstract

**Images:**


Br. J. Cancer (1991), 63, 715-718 © Macmillan Press Ltd., 1991

### Differential expression of neurofilament triplet proteins in carcinoid tumours: an immunohistochemical study

R. Astarloa[1], F. Sanchez-Franco[2], L. Cacicedo & M. Garcia-Villanueva[3]

[1]*Service of Neurology, Jimenez Diaz Foundation; Service of* [2]*Endocrinology and* [3]*Anatomic Pathology, Ramon y Cajal Hospital, Madrid, Spain.*

**Summary** Neurofilaments (NFs) are specific intermediate filaments to neural cells. Mammalian NFs are protein triplets composed of three major subunits with respective molecular weights of approximately 70, 150 and 200 kD. Using an immunohistochemical method, 13 carcinoid tumours from different sites were examined for the presence of these three subunits by means of monospecific antisera. All tumours contained cells that were positive for the 70 Kd subunit; nine cases contained cells immunoreactive for the 150 Kd subunit and only three of them for the 200 kD subunit. The results indicate that the 70 kD subunit is a good overall marker of carcinoid tumours. The 150 and 200 kD subunits are more likely to be absent in carcinoids, both typical and atypical.

Intermediate filaments (IFs) are ubiquitous cytoskeletal; proteins found in mammalian cells. Five classes of IFs can be identified and each class is restricted to certain cell types (Osborn & Weber, 1986; Steiner & Parry, 1985). The immunohistochemical demonstration of IF proteins in tumour cells is widely recognised as an index of cellular differentiation and this approach is therefore extensively used in the histologic diagnosis of human tumours (Battifora, 1988; Parentes & Rubenstein, 1987; Puts *et al.*, 1986). Coexpression of at least two different classes of IFs may be seen in some types of malignancy, notably among lung tumours (Blobel *et al.*, 1985; Gatter *et al.*, 1986; Lehto *et al.*, 1985; Ramaekers *et al.*, 1987).

Neurofilaments (NFs) are specific intermediate filaments of neurons of the central and peripheral nervous systems. Mammalian NFs are protein triplets composed of three major subunits with respective molecular weights of approximately 70, 150 and 200 kD (Robinson & Anderson, 1988; Schalaepfer, 1987). There are some reports of the clinical application of immunohistochemical detection of NFs to human neuroendocrine tumours (Lehto *et al.*, 1983; Merot *et al.*, 1986; Miettinen *et al.*, 1985; Moll *et al.*, 1986). However emphasis should be placed on the fact that this class of IF consists of three subunits and in most of the studies, in the field of tumour pathology, monospecific antibodies corresponding to each of three subunits have not been used.

The present study was undertaken to investigate the immunohistochemical reactivities to neurofilament triplet proteins (NF-70, NF-150 and NF-200) of carcinoid tumours and to determine whether a differential expression could be demonstrated.

### Materials and methods

A total of 13 patients with carcinoid tumours was studied. The clinical data for these patients are summarised in Table I. Surgically removed samples of the tumours were fixed in 10% buffered formalin, embedded in paraffin in the routine manner and sectioned for immunohistochemical study.

### *Preparation of antisera*

Antisera to the individual NF-polypeptides were raised in New Zealand white rabbits, approximately 1 kg in weight. Purification of neurofilament triplet proteins: NF-70, NF-150 and NF-200, was achieved from pig brain by a modification of the method of Shelanski *et al.* (1973). Aliquots of NF fraction were subjected to preparative sodium dodecylsulfate (SDS)-polyacrylamide gel electrophoresis (7'5% polyacrylamide, Tris-glycine buffer system) (Laemmli, 1970). After brief staining with Coomassie Blue and destaining, the portions of the gel slab containing NF-70, NF-150 and NF-200 were excised. The proteins were eluted from the gel pieces into 90% formic acid and then dialysed against deionised water.

The precipitated protein suspensions were emulsified with complete Freund adjuvant and administered subcutaneously to rabbits. Each animal received three doses of approximately 300 ugr protein each, given 3 weeks apart, and serum was collected 7, 10 and 14 days after the second and third injections.

### *Immunoblotting*

After the completion of electrophoresis, one set of lanes was stained in Coomassie Blue while the other sets were each transferred electrophoretically to nitrocellulose (Towbin *et al.*, 1979). Transfer was carried out for 18 h. The nitrocellulose sheets were incubated sequentially as follows: 5% bovine serum albumin (BSA), 30 min, primary antiserum diluted 1/50–1/250, 15 min, wash buffer (WB), 3–10 min, swine anti-rabbit IgG (Dako, Santa Barbara, CA), 1/50, 15 min, WB, 3–10 min, rabbit peroxidase/antiperoxidase (Dako), 1/50, 15 min, WB, 3–10 min. Bands were developed with diaminobenzidine, 0.5 mgr $ml^{-1}$ in 0.015% hydrogen peroxide. WB consisted of 0.15 M NaCl, 0.05 M Tris, Nonidet P-40, 0.1% BSA and 0.02% sodium azide, pH 8.0. All dilutions were made in WB containing 1% BSA.

### *Immunohistochemistry*

Immunohistochemical staining for the triplet proteins was performed according to Sternberger (1986). The paraffin sections from formalin-fixed material were used for staining after a careful deparaffinisation, through xylene and graded alcohols. Endogenous peroxidase was then inactivated by immersion in 0.3% hydrogen peroxide in phosphate buffer (phosphate-buffered saline 0.2 M, pH 7.4 PBS) for 30 min at room temperature followed by the primary antiserum. The primary antisera, all raised in rabbits, were applied at predetermined optimal dilutions (1/150 for NF-70, 1/200 for NF-150 and 1/100 for NF-200) in PBS for overnight at 4°C in a damp chamber. After washing (0.5 M Tris buffer, pH 7.4), the second layer antibody (swine anti-rabbit Ig G, Dako, Santa Barbara, CA) 1/50 was applied for 30 min at room temperature. Finally after washing, rabbit peroxidase-antiperoxidase (PAP) (Dako) diluted 1/50 was placed on the sections. The specific sites of immunoreaction were developed

Correspondence: R. Astarloa, Servicio de Neurologia, Fundación Jiménez Díaz, Avda. Reyes Católicos, 2., 28040 – Madrid, España.
Received 2 January 1990; and in revised form 7 January 1991.

in a solution of PBS containing 0.0006% hydrogen peroxide and 0.025% diaminobenzidine (Sigma, St Louis, MO, USA). The sections were then dehydrated through graded alcohols and xylene and mounted in a permanent medium (DPx).

Negative controls that were carried out comprised dilution of the primary antiserum and their substitution with non-immune serum. Moreover, preabsorption of the primary antibody with the purified antigen resulted in loss of immunostaining. They were used at concentrations of 100–250 ugr $ml^{-1}$ of antiserum, incubated for 2 h at 25°C and centrifugated at 3,000 *g* for 15 min; the resultant supernatant was incubated in the usual manner for the primary antisera.

As positive controls, cultured neurones of cerebral hemispheres from rat embryos and sections from formalin-fixed rat brain were used. Before incubation with the primary antisera, in a humid chamber for 2 h, cells on coverslips were: (1) washed with PBS for 5 min, (2) fixed with 10% paraformaldehyde in PBS (5 min), (3) washed twice with PBS for 10 min each. Sections from rat brain were processed as tumour sections.

### Results

Light microscopic examination revealed well-circumscribed tumours with small, uniform cells frequently grouped in a trabecular pattern, but in cases 7 and 8 spindle-shaped cells were observed. Three cases (7, 8 and 12) showed focal necrosis as well as mitoses.

Figure 1 shows the results of the immunoblotting method for checking the specificities and characterisation of the antisera. Our antisera to the 70 kD, 150 kD and 200 kD subunits reacted to each antigen (subunit) alone. The controls, in which normal rabbit serum (non-immune serum) or antisera preabsorbed with the corresponding antigens in excess were used, did not show immunoreactivity. In positive controls, neurones showed a strong staining and all antibodies reacted to neuronal perikarya and processes (Figures 2 and 3).

The results of the study on the localisation of neurofilament triplet proteins in 13 carcinoid tumours are presented in Table I. The only normal structures labelled with anti-NF protein antibodies were nerve axons and ganglion cells; these were used as the positive controls. In order to investigate the differential expression of neurofilament subunits, we assessed immunoreactivity in several serial sections. All carcinoids showed positive cells when stained with antibody to the 70 kD subunits; nine tumours contained cells immunoreactive for the 150 kD subunit and only three of them for the 200 kD subunit. In most cases, immunoreactivity was juxtanuclear focal. Staining of the sections showed that the antibodies to the 150 kD and 200 kD subunits do not react with all cells that positively stained with antibody to the 70 kD subunit. Furthermore, in each case, the number of positive cells varied depending on the antibody used. Slices

**Figure 1** The specificities of antisera to the 70 kD, 150 kD and 200 kD subunits were examined by the immunoblotting method. Lane A shows SDS gel (7.5% polyacrylamide) of pig neurofilaments stained with Coomassie Blue. Lanes B, C and D are the results of transfer of Lane A to the nitrocellulose sheets. Lanes B, C and D shows the results of staining by peroxidase-antiperoxidase (PAP) complex using anti-70 kD antiserum, anti-150 kD antiserum and anti-200 kD antiserum respectively. It is noted that each antiserum reacts to each antigen (subunit) alone.

**Figure 2** Photomicrograph of section from rat cerebral hemispheres. Immunohistochemical staining for the 70 kD subunit (with counterstain); a neuron clearly positive is shown. Immunostaining for the 150 and 200 kD subunits showed similar results (original magnification: × 1250).

**Figure 3** Immunohistochemical staining of primary culture from embryonic rat cerebral hemispheres with specific 70 kD antiserum. The cells were cultured for 12 days. Note that only the neurones are immunoreactive (original magnification: × 1250).

**Table I**

| | | | | *Neurofilament triplet proteins* | | |
|---|---|---|---|---|---|---|
| *Case* | *Age* | *Sex* | *Sites* | *70 kD* | *150 kD* | *200 kD* |
| 1 | 42 | M | Appendix | + | + | + |
| 2 | 50 | F | Appendix | + | + | – |
| 3 | 32 | M | Appendix | + | + | – |
| 4 | 40 | F | Intrabronchial | + | + | + |
| 5 | 41 | M | Intrabronchial | + | + | – |
| 6 | 55 | F | Intrabronchial | + | – | – |
| 7 | 62 | F | Intrabronchial | + | – | – |
| 8 | 47 | M | Peripheral lung | + | – | – |
| 9 | 75 | F | Ileum | + | + | – |
| 10 | 70 | M | Ileum | + | + | – |
| 11 | 71 | F | Gallbladder | + | + | + |
| 12 | 58 | M | Mediastinum | + | – | – |
| 13 | 64 | F | Breast | + | + | – |

stained with antibody to the 70 kD subunit showed the largest number of positive cells; there were fewer cells reacting with antibody to the 150 kD subunit and even fewer cells reacting with antibody to the 200 kD subunit. Figures 4–6 illustrate these findings.

**Figure 4** Immunohistochemical staining of a carcinoid tumour with monospecific antibodies to neurofilament triplet proteins. (PAP method). Immunostaining for the 70 kD subunit with counterstain (original magnification: × 1,000).

**Figure 5** Immunohistochemical staining of a carcinoid tumour with monospecific antibodies to neurofilament triplet proteins. (PAP method). Immunostaining for the 150 kD subunit with counterstain (original magnification: × 1,000). Note that a greater number of immunoreactive cells is found with antibody to the 70 kD subunit.

**Figure 6** Immunohistochemical staining of a carcinoid tumour with antibody to the 200 kD subunit (PAP method). Note the scarcity of immunoreactive cells (original magnification: × 1,000).

At times, the staining reactions were weaker with antibody to the 200 kD subunit. It was possible to detect tumour cells in which the 70 kD subunit existed by itself, but tumour cells in which the 150 or 200 kD subunits existed alone could not be detected. Positive cells for both 150 and 200 kD subunits were not demonstrated in all atypical carcinoids as well as in one of the 10 typical tumours.

### Discussion

In this study, we analysed immunohistochemically the differential expression of neurofilament triplet proteins (NF-70, NF-150 and NF-200) in 13 carcinoid tumours from different sites. Three tumours were classified as atypical carcinoids, two bronchopulmonary and one in the mediastinum.

Neurofilaments (NFs) are specific neural markers and have been found in some neuroendocrine neoplasms: oat cell carcinoma of the lung (Broers *et al.*, 1985; Lehto *et al.*, 1983), islet cell tumours and cutaneous neuroendocrine carcinoma (Domagala *et al.*, 1987; Merot *et al.*, 1986). In relation to carcinoids, our results are in agreement with early reports (Altmannsberger *et al.*, 1984; Lehto *et al.*, 1984; Lehto *et al.*, 1985). In contrast, some authors (Broers *et al.*, 1985; Trojanowski *et al.*, 1984) considered carcinoid tumours to be negative for neurofilament antigens; Blobel *et al.* (1985) found weak immunoreactivity for the 70 kD subunit in two of four bronchopulmonary carcinoids examined by immunofluorescence microscopy. Some factors such as tissue preparation (e.g., interval from excision to fixation, duration of fixation, embedding methods, immunohistochemical procedure) may have contributed to the divergent results. Also it is possible that the anti-NF monoclonal antibodies employed in some studies do not recognise the epitopes of NFs present in the tumours of putative neuronal origin. Christen *et al.* (1987), using monoclonal antibodies for phosphorylated and non-phosphorylated isoforms of the high-molecular-weight neurofilament subunit, detected both NF-proteins in typical bronchial carcinoids but not in atypical carcinoids. We found neither immunoreactive cells for the 200 kD subunit in tumours with atypical histological characteristics. The presence of NF-immunoreactivity in juxtanuclear aggregates was striking; masses of NFs have been described in a variety of pathologic conditions and they may reflect abnormalities in intermediate filament metabolism (Trojanowski, 1987).

The histogenesis of the dispersed neuroendocrine system remains uncertain (Gould *et al.*, 1983). Many neuroendocrine carcinomas express more than one class of intermediate filaments, often coexpress cytokeratin and NFs (Gatter *et al.*, 1986; Lehto *et al.*, 1985; Merot *et al.*, 1986; Miettinen *et al.*, 1985; Ramaekers *et al.*, 1987) and this coexpression may be related to a special histogenesis. Tumours with an established neural crest derivation display divergence in their intermediate filament expression, probably reflecting the early segregation of neural crest cells before migration and their different susceptibility to environmental influences (Ziller *et al.*, 1983). In bronchopulmonary carcinoids, Kulchitsky cells are considered to be candidate precursor cells for these tumours and it has reported the presence of NF-subunits in Kulchitsky cells of human bronchial epitheliam (Torika *et al.*, 1986); it will be important to learn whether these cells express keratin filament proteins. Some authors (Fischer *et al.*, 1989; Van Muijen *et al.*, 1984) have detected immunoreactive cells for NF-proteins in circumscribed areas of some squamous cell carcinomas, but it seems unrelated to the morphologic differentiation of the neoplasms.

The present study is the first in which the differential expression of neurofilament triplet proteins was evaluated in carcinoids. Our results revealed that the 70 kD subunit is a good indicator of this tumour both from the aspect of the number of patients with positive findings and the number of immunoreactive cells; the 150 and 200 kD subunits are more likely to be absent in carcinoids, both typical and atypical. However only 13 cases have been included in this series and more definitive conclusions must await further studies.

### References

ALTMANNSBERGER, M., OSBORN, M., DROESE, M., WEBER, K. & SCHANER, A. (1984). Diagnostic value of intermediate filament antibodies in clinical cytology. *Klin Wochenschr.*, **62,** 114.

BATTIFORA, H. (1988). Clinical applications of the immunochemistry of filamentous proteins. *Am. J. Surg. Pathol.*, **12** (Suppl. 1), 24.

BLOBEL, G.A., GOULD, V.E. & MOLL, R. (1985). Coexpression of neuroendocrine markers and epithelial cytoeskeletal proteins in bronchopulmonary neuroendocrine neoplasms. *Lab. Invest.*, **52,** 39.

BROERS, J.L., CARMY, D.N., DE LEY, L., VOONS, G.P. & RAMAEKERS, F.C.S. (1985). Differential expression of intermediate filament proteins distinguishes classic from variant small-cell lung cancer cell lines. *Proc. Natl Acad. Sci. USA*, **98,** 4409.

BROERS, J., HUYSMANS, A. & MOESKER, O. (1985). Small cell lung cancers contains intermediate filaments of the cytokeratin type. *Lab. Invest.*, **52,** 113.

CHRISTEN, B., TROJANOWSKI, J.Q. & PIETRA, G.G. (1987). Immunohistochemical demonstration of phosphorylated and non-phosphorylated forms of human neurofilament subunits in human pulmonary carcinoids. *Hum. Pathol.*, **18,** 997.

DOMAGALA, W., LUBINSKI, J., LASOTA, J., GIRYN, I., WEBER, K. & OSBORN, N. (1987). Neuroendocrine (Merkel-Cell) carcinoma of the skin; cytology, intermediate filament typing and ultrastructure of tumour cells in fine needle aspirates. *Acta Cytol.*, **31,** 267.

FISCHER, H.P., WALLNER, F., MAIER, H., WEBER, K., OSBORN, M. & ALTMANNSBERGER, M. (1989). Coexpression of intermediate filaments in squamous cell carcinomas of upper aerodigestive tract before and after radiation and chemotherapy. *Lab. Invest.*, **61,** 433.

GATTER, K.C., DUNNILLS, M.S., VAN MUIJER, G.N.P. & MASON, D.Y. (1986). Human lung tumours may coexpress different classes of intermediate filaments. *J. Clin. Pathol.*, **39,** 950.

GOULD, V.E., LINNOILA, R.I., MEMOLI, V.A. & WARREN, W.H. (1983). Neuroendocrine components of the bronchopulmonary tract: hyperplasias, dysplasia and neoplasm. *Lab. Invest.*, **49,** 519.

LAEMMLI, H.K. (1970). Cleavage of structural proteins during the assembly of the head of bacteriophage $T_4$. *Nature*, **227,** 680.

LEHTO, V.P., STENMAN, S., MIETTINEN, M., DAHL, D. & VIRTANEN, I. (1983). Expression of a neural type of intermediate filament as a distinguishing feature between oat cell carcinoma and other lung cancers. *Am. J. Pathol.*, **110,** 113.

LEHTO, V.P., MIETTINEN, M., DAHL, D. & VIRTANEN, I. (1984). Bronchial carcinoid cells contain neural-type of intermediate filaments. *Cancer*, **54,** 624.

LEHTO, V.P., MIETTINEN, M. & VIRTANEN, I. (1985). A dual expression of cytokeratin and neurofilaments in bronchial carcinoid cells. *Int. J. Cancer*, **35,** 421.

MEROT, Y., MARGOLIS, R.J., DAHL, D., SAURAT, J.H. & MIHM, M.C. (1986). Coexpression of neurofilament and keratin proteins in cutaneous neuroendocrine carcinoma cells. *J. Invest. Dermatol.*, **86,** 74.

MIETTINEN, M., LEHTO, V.P., DAHL, D. & VIRTANEN, I. (1985). Varying expression of cytokeratin and neurofilaments in neuroendocrine tumours of human gastrointestinal tract. *Lab. Invest.*, **52,** 429.

MOLL, R., OSBORN, M., HARTSCHUH, W., MOLL, I. & MAHRLE, G. (1986). Variability of expression and arrangement of cytokeratin and neurofilaments in cutaneous neuroendocrine carcinomas: immunohistochemical and biochemical analysis of 12 cases. *Ultrastruct. Pathol.*, **10,** 473.

OSBORN, M. & WEBER, K. (1986). Intermediate filament proteins: a multigene family distinguishing major cell lineages. *Trends Biol. Sci.*, **11,** 469.

PUTS, J.J.G., VOOIJS, G.P., HUYSMANA, A., VAN ASPERT, A. & RAMACHERS, F.C.S. (1986). Cytoskeletal proteins as tissue-specific markers in cytopathology. *Exp. Cell Biol.*, **54,** 73.

PERENTES, E. & RUBINSTEIN, L.J. (1987). Recent applications of immunoperoxidase histochemistry in human neuro-oncology: an update. *Arch. Pathol. Lab. Med.*, **111,** 796.

RAMAEKERS, F., BROERS, J., KLEIN-ROT, M., OOSTENDORP, T., WAGENAAR, S. & VOOIJS, P. (1987). Detection of epithelial – and neural type of intermediate filament proteins in human lung tumours. *Acta Histochem.*, **34,** S45.

ROBINSON, P.A. & ANDERSON, B.H. (1988). Neurofilament Probes. A review of neurofilament distribution and biology. *Rev. Neurosci.*, **2,** 1.

SCHALAEPFER, W.W. (1987). Neurofilaments: structure, metabolism and implications in disease. *J. Neuropathol. Exp. Neurol.*, **46,** 117.

SHELANSKI, M.L., GASKIN, F. & CANTOR, C.R. (1973). Microtubule assembly in the absence of added nucleotides. *Proc. Natl Acad. Sci. USA*, **70,** 765.

STEINER, P.M. & PARRY, D.A.D. (1985). Intermediate Filaments. *Ann. Rev. Cell*, **1,** 441.

STERNBERGER, L.A. (1986). *Immunocytochemistry*, 3rd Ed. Wiley, J. (ed.). New York.

TORIKA, C., MUKA, M. & KAWAKITA, H. (1986). Neurofilaments of Kultschitsky cells in human lung. *Acta Pathol. Jpn.*, **36,** 93.

TOWBIN, H., STAEHELIN, T. & GORDON, J. (1979). Electrophoretic transfer of proteins from polyacrylamide gels to nitrocellulose sheets: procedure and some applications. *Proc. Natl Acad. Sci. USA*, **76,** 4350.

TROJANOWSKI, J.Q., LEE, V.M. & SCHALAEPFER, W.W. (1984). An immunohistochemical study of human central and peripheral nervous system tumours using monoclonal antibodies against neurofilaments and glial filaments. *Hum. Pathol.*, **15,** 248.

TROJANOWSKI, J.Q. (1987). Neurofilament proteins and human nervous system tumours. *J. Histochem. Cytochem.*, **35,** 999.

VAN MUIJEN, G.N.P., RUITER, D.J., VAN LEEUWEN, C., PRINS, F.A. & WARNAAR, S.O. (1984). Cytokeratin and neurofilament in lung carcinomas. *J. Pathol.*, **116,** 363.

ZILLER, C., DUPIN, E., BRAZEAN, P., PAULIN, D. & LEDONARIN, N.M. (1983). Early segregation of a neural precursor cell line in the neural crest as revealed by culture in a chemically defined medium. *Cell*, **32,** 627.